# Gestation Impeded by Encystead Dropsy—Relief by Paracentesis

**Published:** 1844-10-19

**Authors:** 


### RECORD OF MEDICAL SCIENCE.

### GESTATION IMPEDED BY ENCYSTEAD DROPSY—RELIEF BY PARACENTESIS.

BY DR. HANDYSIDE.

The patient had borne three children, and had three miscarriges. During each successive pregnancy the enlargement, which was situated on the left side of the abdomen, increased, and when Dr. H. was called, presented the characters of ovarian dropsy. On the last occasion, after completing her fifth month of gestation, she was repeatedly attacked by urgent and protracted fits of dyspnœa, produced by the enormous distention of the abdomen by the dropsical swelling, and there was imminent risk of an immediate miscarriage; upon this it was resolved by Drs. Sidey, Pagan, and Handyside, who consulted together on the case, to afford the patient the temporary benefit of the operation of paracentesis, notwithstanding that the prognosis generally was so very unfavourable.

The fluid removed resembled that of ovarian dropsy, being of a viscid albuminous consistence, very tenacious on cooling, solidified under nitric acid and heat. It was of a dark brown colour, and presented a trace of arterial blood, as if it had flowed from a very vascular cyst. Two and a half gallons was the quantity removed: and the patient became much relieved. She went on favourably till the thirteenth day after the operation, when she had the apprehended miscarriage, and sunk from the separation of the placenta, bleeding, and general exhaustion, before Dr. Sidey was called.

On examination of the body, the fibro-serous cyst, which, previous to recourse being had to the trocar, had lain in contact with the anterior wall of the abdomen, was found to have receded into the left hypochondriac region, between the cardiac end of the stomach, the diaphragm, and the left angle of the colon, within the lesser sac of the peritoneum. It was found partially refilled, and on being incised discharged nearly a gallon of dark coloured fluid similar to that removed by operation.

The cyst, on its interior, presented well marked encephaloid tumours, which had protruded from its middle or fibrous coat. The stomach presented, underneath its mucous lining, two patches of the cerebriform deposit; minute tumours of the same character were situated on different parts of the anterior wall of the peritoneum; and the liver, which had attained three times its natural weight, presented the carcinomatous, mingled with the hematoid degeneration. Ascites, to the amount of one or two pounds, also existed, and was demonstrated by Dr. H. to be the effect of the three-fold alteration of structure. The pelvic viscera (with the exception of the ordinary traces of very recent abortion, including separation of the fœtal membranes,) and the thoracic viscera, were healthy.—*Lond. and Edin. Monthly Journ. Med. Science.*

### DR. CORMACK ON IPECACUAN AS A COUNTER-IRRITANT.

Dr. Cormack detailed the result of his hospital experience of ipecacuan as a counter-irritant in the form of ointment, as recommended by Dr. Hannay of Glassgow. Dr. C. had tried the ipecacuan in a great many cases, and in the proportion of 10 to 12 of them it had failed to produce any eruption, even when the powder was in the proportion of half an ounce to an ounce of lard. In a few persons only, with a delicate skin, or who had had recent blisters on the surface experimented on, did he succeed in bringing out an eruption. The eruption was vascular in three cases which were carefully observed. In the same persons on whose skin the ointment produced no effect, a good crop of pustules was in every instance brought out by one, two, or three frictions with a liniment of equal parts of olive and croton oil. Dr. C. believed that there were many vegetable powders which would be found more active counter-irritants than ipecacuan.

Dr. Handyside and Dr. Douglas Maclagan stated that they had met with no good result from its employment. Their experience corroborated that of Dr. C.—*Ibid.*

